# Incorporating genetic networks into case-control association studies with high-dimensional DNA methylation data

**DOI:** 10.1186/s12859-019-3040-x

**Published:** 2019-10-22

**Authors:** Kipoong Kim, Hokeun Sun

**Affiliations:** Department of Statistic, Pusan National University, Busan, 46241 Korea

**Keywords:** DNA methylation, Genetic network, Regularization, Dimension reduction

## Abstract

**Background:**

In human genetic association studies with high-dimensional gene expression data, it has been well known that statistical selection methods utilizing prior biological network knowledge such as genetic pathways and signaling pathways can outperform other methods that ignore genetic network structures in terms of true positive selection. In recent epigenetic research on case-control association studies, relatively many statistical methods have been proposed to identify cancer-related CpG sites and their corresponding genes from high-dimensional DNA methylation array data. However, most of existing methods are not designed to utilize genetic network information although methylation levels between linked genes in the genetic networks tend to be highly correlated with each other.

**Results:**

We propose new approach that combines data dimension reduction techniques with network-based regularization to identify outcome-related genes for analysis of high-dimensional DNA methylation data. In simulation studies, we demonstrated that the proposed approach overwhelms other statistical methods that do not utilize genetic network information in terms of true positive selection. We also applied it to the 450K DNA methylation array data of the four breast invasive carcinoma cancer subtypes from The Cancer Genome Atlas (TCGA) project.

**Conclusions:**

The proposed variable selection approach can utilize prior biological network information for analysis of high-dimensional DNA methylation array data. It first captures gene level signals from multiple CpG sites using data a dimension reduction technique and then performs network-based regularization based on biological network graph information. It can select potentially cancer-related genes and genetic pathways that were missed by the existing methods.

**Electronic supplementary material:**

The online version of this article (10.1186/s12859-019-3040-x) contains supplementary material, which is available to authorized users.

## Background

In human genetic association studies, statistical methods that can incorporate genetic network information into association analysis have been widely used since the seminal paper of Li and Li [1]. In Crohn’s disease association study, for instance, Chen et al. [2] have demonstrated that neighboring genes within a genetic pathway tend to have similar association patterns. Zhang et al. [3] utilized human protein-protein interaction network to identify gene expression features associated with ovarian cancer. Kim et al. [4] developed a new prognostic scoring system for breast cancer patients based on six large genetic network databases. Ren et al. [5] combined the cell cycle pathway and p53 signaling pathway to identify important genes for analysis of Type 2 diabetes mellitus. When genes are functionally related to each other in a genetic network, statistical methods utilizing prior biological network knowledge indeed outperform other methods that ignore the genetic network structures.

In methodological research, network-based regularization proposed by Li and Li [1, 6] have shown promising selection results for analysis of high-dimensional gene expression data. It basically combines the *l*_1_-norm penalty and the squared *l*_2_-norm penalty with a Laplacian matrix representing a graph structure among genes so that both sparsity and smoothness among biologically linked genes can be induced. Although the original network-based regularization was limited to a linear regression model where an outcome variable is quantitative, it has been extended to case-control association study replacing a least square loss function by a negative logistic likelihood [5, 7]. A conditional logistic likelihood and a partial Cox likelihood were also used for 1:1 matched case-control analysis and censored survival analysis, respectively [3, 8–10]. One noticeable advantage of network-based regularization is computational efficiency due to convex optimization. That is to say, variable selection can be conducted with relatively fast computational speeds even for high-dimensional genomic data, as we adopt one of the well-designed computational algorithms such as cyclic coordinate descent and gradient descent algorithms [11–14].

However, network-based regularization has been mainly applied to gene expression data where an individual gene is considered as one predictor in a regression framework. Suppose that we have gene expression data with *p* genes. In a given biological graph where a node represents a gene and an edge represents a genetic link between two genes, network-based regularization can employ the *p*-dimensional Laplacian matrix to select outcome-related genes based on the biological network structure. In recent association studies on epigenetics, relatively many statistical methods for analysis of high-dimensional DNA methylation data have been proposed to identify cancer-related CpG sites and their corresponding genes [7, 8, 15–18]. But, most of these methods are not designed to utilize genetic network information in epigenome-wide association studies. Network-based regularization cannot be directly applied to high-dimensional DNA methylation data because an individual CpG site is considered as one predictor and one single gene consists of multiple CpG sites. In other words, the dimension of the Laplacian matrix representing a biological network does not match with that of DNA methylation data.

In this article, we propose new approach that incorporates biological network information into case-control association analysis with high-dimensional DNA methylation data. The proposed approach combines one of data dimension reduction techniques with network-based regularization to identify outcome-related genes, given a biological network. We considered four different dimension reduction techniques, which are principal component (PC), normalized principal component (nPC), supervised principal component (sPC), and partial least square (PLS). The proposed approach first captures gene-level signals from multiple CpG sites using one of dimension reduction techniques and then regularizes them to perform gene selection based on the biological network. We performed extensive simulation studies where the performance of four dimension reduction techniques was compared with each other, and the proposed approach was also compared with other statistical methods that ignore network information, including group lasso and commonly used individual group-based tests. Finally, we investigated the correlation patterns of high-dimensional DNA methylation data from four breast invasive carcinoma cancer subtypes, and found that DNA methylation levels among linked genes in a biological network are indeed highly correlated with each other. The proposed approach was then applied to 450K DNA methylation data to identify potentially cancer-related genes and genetic pathways, incorporating seven large genetic network databases.

## Results

### Simulation studies

In order to simulate methylation data where linked genes within a biological network graph are correlated with each other, a three-step process was conducted. In step 1, we made the *p*-dimensional covariance matrix from an arbitrary graph based on a Gaussian graphical model. In step 2, *p* latent variables were generated from two different multivariate normal distributions that have the same covariance but a different mean vector. In step 3, methylation values for both neutral and outcome-related CpG sites were simulated based on each of latent variables.

**Fig. 1:**
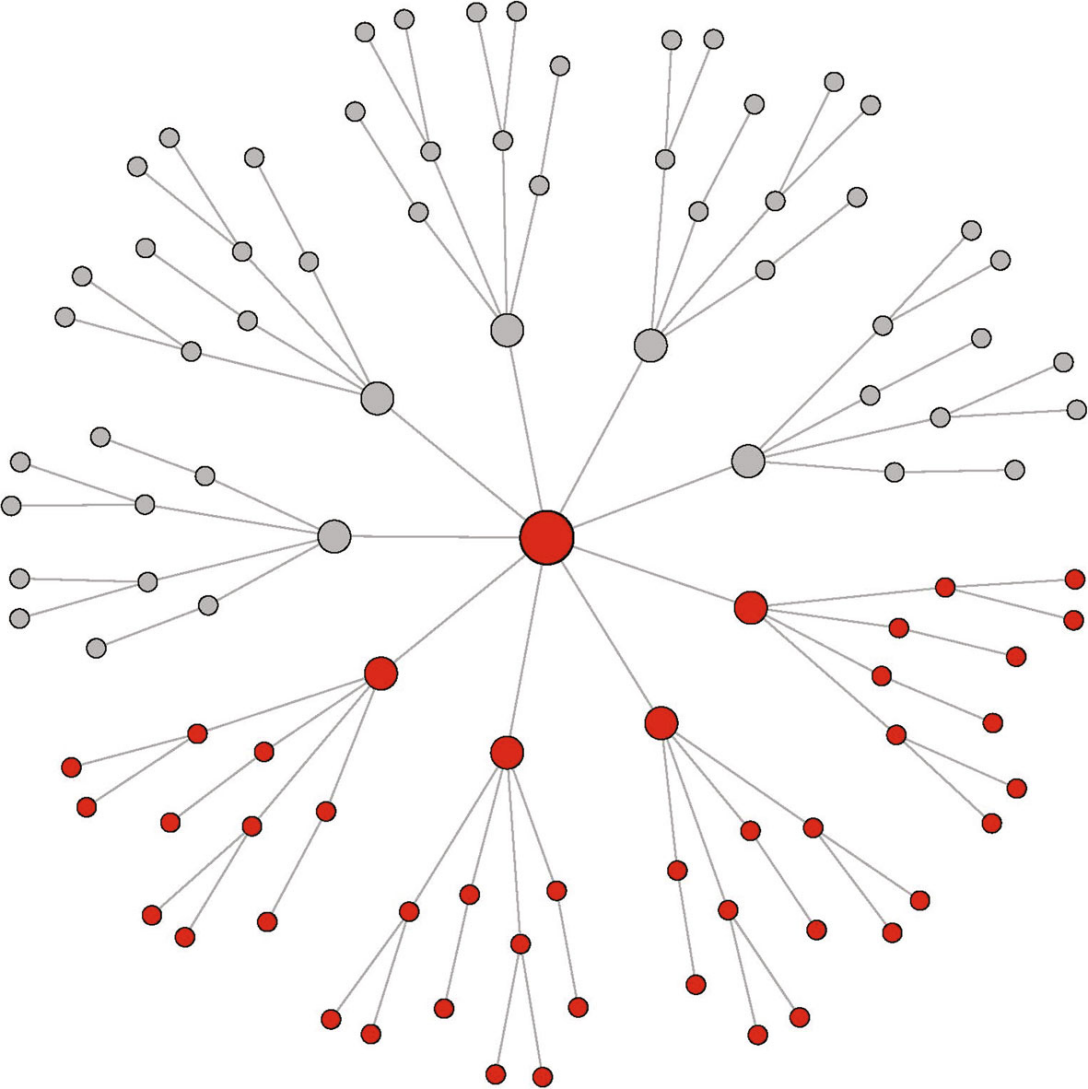
An example of a network module used in simulation studies. It has a total of 100 genes, where the colored 45 genes are assumed to be outcome-related genes and consist of one centered gene plus four different groups of 11 genes

Specifically, we first created an arbitrary network graph in Fig. 1 to mimic a biological network that contains a hub gene plus many other genes with a few links. We assumed that we have 10 disjointed network modules each of which consists of 100 genes corresponding to the network in Fig. 1. That is, we have a total of *p*=1000 genes. In the first scenario, we further assumed that only 45 genes in the first network module are outcome-related and the remaining 9 network modules do not include outcome-related genes. Figure 1 depicts these 45 colored genes out of 100 genes in the first network module. They consist of one centered genes with four groups of linked genes. We denote these four groups of outcome-related genes as *g*_1_,*g*_2_,*g*_3_, and *g*_4_, respectively.

The difference between 45 outcome-related genes and the remaining 955 neutral genes were distinguished by two different mean vectors between cases and controls. The mean vector of the control group is fixed as 0, while the mean vector of case group is defined as ***μ***=(*μ*_1_,*μ*_2_,…,*μ*_*p*_)^T^. For 995 neutral genes, we set *μ*_*j*_=0 so that there is no mean difference between cases and controls. In contrast, if the *j*-th gene is one of the 45 outcome-related genes, *μ*_*j*_ is defined as

$$\mu_j \sim \left\{\begin{array}{ll} \delta & \, \text{if \,centered gene} \\ \frac{\delta}{3}\sqrt{d_j} & \, \text{if} \,\, j \in g_1 \text{ or} j \in g_3 \\ -\frac{\delta}{3}\sqrt{d_j} & \, \text{if} \,\, j \in g_2 \text{ or} j \in g_4, \\ \end{array}\right. $$

where *δ* is the strength of association signals and *d*_*j*_ is the total number of genetic links for the *j*-th gene. We set *δ*=1.5 so that |*μ*_*j*_| ranges from 0.5 to 1.5. Note that in our simulation a gene with more genetic links can have stronger signals than a gene with less links. Also, genes in the same network module can be either positively or negatively associated with an outcome.

Next, we applied a Gaussian graphical model [19] to generate a covariance matrix of 1000 genes, where the linked genes are correlated with each other according to the network structure in Fig. 1. The key assumption of the Gaussian graphical model is that non-zero entries of an inverse covariance matrix imply genetic links between two genes [20, 21]. Therefore, the correlation between linked genes are much higher than that of unlikend genes. In our example, the inverse covariance matrix corresponding to our 10 network modules is very sparse since the number of links for an individual gene is at most 9. More detailed procedure to generate a covariance matrix given a network graph is described by [20]. Let us denote the generated covariance matrix by *Σ*.

In our simulation, we assumed that the covariance is the same between cases and controls while the mean vector is different from each other. The *p*-dimensional latent variable of the *i*-th individual *z*_*i*_ was then simulated from two different multivariate normal distributions such that

$$z_i \sim \left\{\begin{array}{ll} N(0, \Sigma) & \,\text{if the \text{i}-th individual is control} \\ N(\boldsymbol{\mu}, \Sigma) & \,\text{if the \text{i}-th individual is case} \end{array}\right. $$

where *z*_*i*_=(*z*_*i*1_,…,*z*_*ip*_)^T^ and *z*_*im*_ represents the latent value of the *m*-th gene of the *i*-th individual. Based on these latent values, we finally generated methylation data assuming each gene consists of 10 CpG sites. That is, we additionally generated methylation values of 10 CpG sites each gene so that our simulation data has a total of 10,000 CpG sites. The methylation value of the *i*-th individual and the *j*-th CpG site in the *m*-th gene is denoted by $x_{ij}^{[m]}$, which was generated from

$$x_{ij}^{[m]}=\left\{\begin{array}{cl} z_{im} + \epsilon_{ij}, & j=1,\ldots, \omega \\ \bar{\epsilon}_{ij}, & j=\omega+1,\ldots,10 \\ \end{array} \right. $$

where *ε*_*ij*_∼*N*(0,*σ*^2^) and $\bar {\epsilon }_{ij} \sim N \left (\frac {1}{n}\sum _{i=1}^{n} z_{im}, \sigma ^{2}\right)$. We have two parameters to vary the simulation setting. The first one is *ω* that is the total number of CpG sites correlated with the latent value. It essentially controls the number of causal/neutral CpG sites in the outcome-related gene. The other one is an error variance, *σ*^2^ which controls the noise level of association signals. The sample size was 200 consisting of 100 cases and 100 controls.

In the first comparison, we considered five regularization methods where four methods used the same network-based regularization but combined with one of four reduction techniques which are principal components (Net+PC), normalized principal components (Net+nPC), supervised principal components (Net+sPC), and partial least squares (Net+PLS), respectively. As described in “Materials and methods” section, each method first captures gene level signals from 10 CpG sites of individual genes, and then applies the network-based regularization utilizing the pre-specified network graph information in Fig. 1. The other comparing method is group lasso which performs gene selection without using genetic network information [22, 23].

**Fig. 2:**
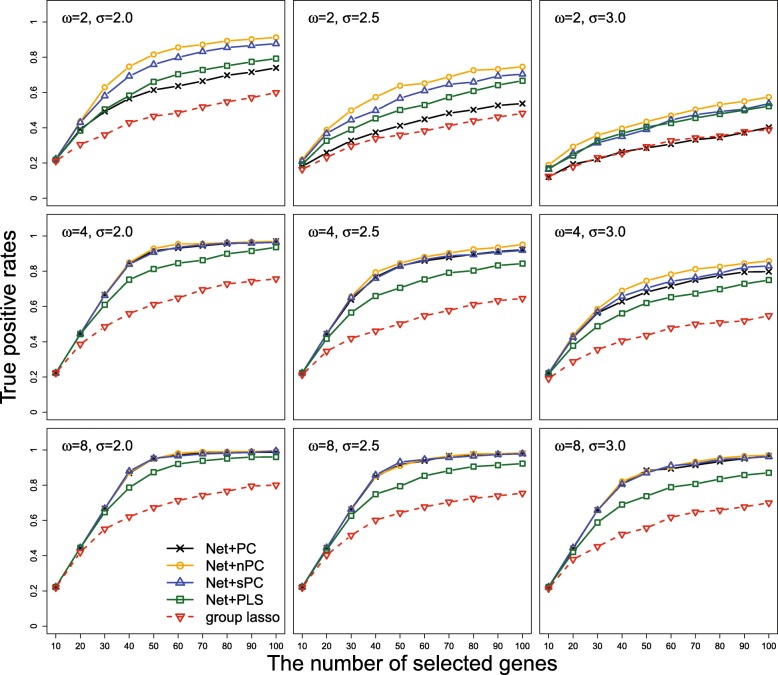
The averaged true positive rates of the network-based regularization methods combined with four different dimension reduction techniques such as principal components (Net+PC), normalized PC (Net+nPC), supervised PC (Net+sPC), partial least square (Net+PLS) and group lasso are displayed along with different number of selected genes ranked by selection probability, when the number of causal CpG sites in an outcome-related gene *ω* and the noise level *σ* have different values

The selection performance of five methods were evaluated based on true positive rate (TPR) which is equivalent to the number of selected genes among 45 outcome-related genes divided by 45. Since the TPR result depends on the total number of selected genes, we compared TPRs of five methods when they selected the exact same number of genes. Note that false positive rates of five selection methods in our simulation is inversely proportional to TPR, because comparisons were made when the number of outcome-related genes was fixed as 45 and the same number of genes was selected by all methods. Therefore, higher TPR clearly indicates a better method when five methods select the exactly same number of genes. Each method first computed selection probabilities of individual genes and then top 10,20,…,100 genes were ranked by their selection probabilities. In Fig. 2, the averaged TPRs of five methods over 100 simulation replications are displayed along with different number of selected genes when *ω*=2,4 or 8, and *σ*=2.0,2.5 or 3.0.

In Fig. 2, it is noticeable that group lasso shows the worst selection performance in all of nine simulation settings. This indicates that utilizing genetic network information indeed improves selection performance when methylation data are highly correlated among linked genes. Also, we can see that combining with partial least square is not appropriate since it has relatively lower TPR than combining with other dimension reduction techniques. When the number of causal CpG sites in a gene is large (*ω*=8), three methods such as Net+PC, Net+nPC and Net+sPC have almost the same TPR regardless of the size of the error variance. However, TPRs of Net+nPC is better than those of Net+PC and Net+sPC when the number of causal CpG sites in a gene is less than 8. Particularly, Net+PC shows very poor selection performance when *ω*=2. Although Net+sPC is much better than Net+PC, it has slightly lower TPR than Net+nPC when *ω*=2. It seems that Net+nPC shows the best selection performance in all simulation settings. Consequently, we can conclude that the normalized principal component is the most appropriate feature to represent multiple CpG sites from each gene, compared with other dimension reduction techniques.

**Fig. 3:**
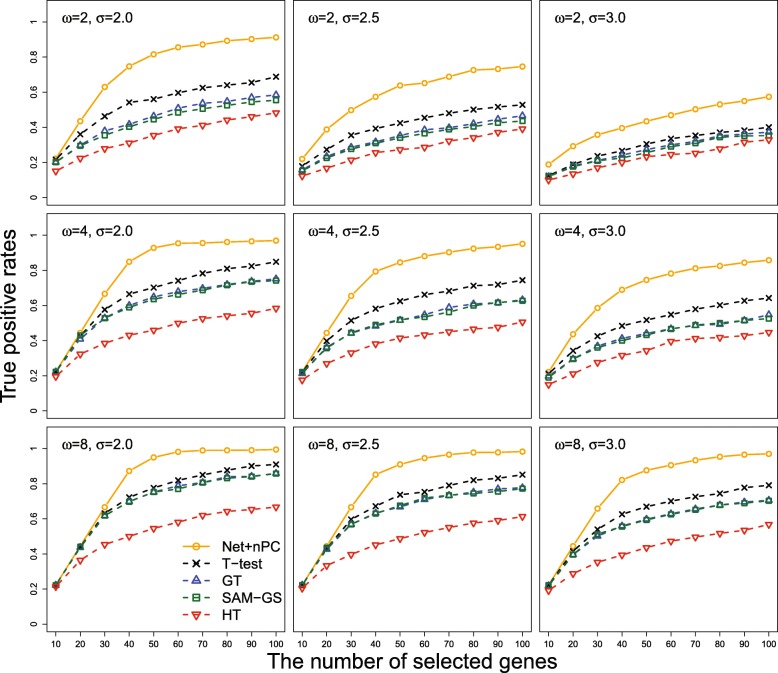
The averaged true positive rates of the network-based regularization method combined with normalized principal component (Net+nPC), two sample *t*-test using PCA (T-test), global test (GT), SAM-GS and Hotelling’s *T*^2^ test (HT) are displayed along with different number of selected genes ranked by selection probability for Net+nPC and *p*-values for four individual tests, when the number of causal CPG sites in an outcome-related gene *ω* and the noise level *σ* have different values

In the next comparison, we considered commonly used gene-based hypothesis tests where each gene is tested one at a time so the *p*-values of 1000 genes were simultaneously computed. Since results from hypothesis testing and variable selection are difficult to directly compare with each other, we ranked genes by *p*-values from each test and selected a particular number of top ranked genes by p-values like 10,20,…,100. The TPRs of these top ranked genes were compared with those of genes ranked by selection probabilities from Net+nPC, which shows the best selection performance among 5 regularization methods. Since each gene consists of 10 CpG sites, we considered four representative group-based tests such as two sample *t*-test based on PCA, global test [24], SAM-GS [25], and Hotelling’s *T*^2^ test [26]. In Fig. 3, the averaged TPRs of five methods over 100 simulation replications are displayed along with different number of selected genes when *ω*=2,4 or 8, and *σ*=2.0,2.5 or 3.0. In Fig. 3, we can see that Net+nPC overwhelms four individual tests in all of nine simulation settings. Since individual group tests also do not utilize network graph information, they are not comparable with the proposed method. The numerical values of TPRs of 4 individual tests and 5 regularization methods are summarized in Table 1 when all methods selected top 50 genes.

**Table 1:** The averaged true positive rates of 4 individual tests and 5 different regularization methods when each method selected top 50 genes

	*ω*=2			*ω*=4			*ω*=8		
Method	*σ*=2.0	*σ*=2.5	*σ*=3.0	*σ*=2.0	*σ*=2.5	*σ*=3.0	*σ*=2.0	*σ*=2.5	*σ*=3.0
T-test	0.5595	0.4243	0.3043	0.7016	0.6263	0.5167	0.7767	0.7376	0.6689
GT	0.4662	0.3538	0.2738	0.6492	0.5176	0.4414	0.7557	0.6698	0.6002
SAM-GS	0.4452	0.3405	0.2548	0.6358	0.5186	0.4318	0.7529	0.6765	0.5964
HT	0.3519	0.2700	0.2310	0.4566	0.4118	0.3394	0.5420	0.4847	0.4351
group lasso	0.4644	0.3536	0.2842	0.6089	0.4959	0.4272	0.6697	0.6374	0.5487
Net+PLS	0.6592	0.4963	0.3969	0.8106	0.7020	0.6148	0.8733	0.7910	0.7322
Net+PC	0.6136	0.4078	0.2777	0.9141	0.8276	0.6762	0.9504	0.9145	0.8801
Net+sPC	0.7592	0.5663	0.3862	0.9066	0.8283	0.7004	0.9547	0.9310	0.8644
Net+nPC	0.8148	0.6376	0.4338	0.9276	0.8456	0.7455	0.9504	0.9103	0.8760

In the second scenario of the simulation study, we assumed that 48 genes among 1000 are outcome-related, where 12 genes from each of four network modules are only outcome-related. So, the remaining 6 modules do not include outcome-related genes. Additional file 1 depicts 48 colored genes in the four network modules. The outcome-related genes in each network module consists of one centered gene with 11 linked genes. Similar to the first scenario, we assumed that 24 genes in two modules are positively associated with an outcome, while the remaining genes in the other modules are negatively associated with an outcome. All other simulation settings such as how to generate the mean vector and the covariance matrix, data dimension and sample size were not changed. The TPRs of the network-based regularization incorporated with nPC were also compared with those of four other regularization methods and those of four individual tests in Additional files 2 and 3, respectively. In this scenario, the Net+nPC is still superior to all other methods in terms of true positive rates of selected genes.

Finally, we generated another simulation data where each gene includes a different number of CpG sites. That is, we considered both big and small genes in this simulation while the first two scenarios assumed that all genes have 10 CpG sites. The number of CpG sites each gene was simulated from a Gamma distribution for all of *p*=1000 genes. We found that the distribution of the number of CpG sites from our breast cancer data is similar to a Gamma distribution. The histograms of the number of CpG sites each gene for both simulation data generated from a Gamma distribution and breast cancer data are displayed in Additional file 4. Since big genes can have a greater number of causal CpG sites than small genes, we assumed that 40% of CpG sites within 45 outcome-related genes are causal sites and the error variance was fixed as 2.5. The TPRs of 4 individual tests and 5 regularization methods are shown in Additional file 5. In this simulation, Net+nPC still outperforms all other methods.

### Analysis of breast cancer data

We applied the proposed method to the case-control type of 450K DNA methylation datasets of four subtypes of breast invasive carcinoma (BRCA) from TCGA project [18, 27]. We conducted standard quality control steps where sites on sex chromosomes, sites with missing values and sites overlap with known single nucleotide polymorphisms were first removed out and type I/II probe bias was then corrected using the ’wateRmelon’ package. After pre-processing, the dataset ended up with 317,487 CpG sites over 19,296 genes for 59 independent normal samples and 187 tumor samples which contain 31 samples for the Basal-like subtype, 12 for the Her2 subtype, 99 for the LumA subtype and 45 for the LumB subtype. Therefore, we could conduct four different case-control association studies where tumor samples from four different subtypes were regarded as a case group and the same normal samples were considered as a control group. In order to utilize biological network information, we employed an R package ‘graphite’ which combined 7 genetic network databases from Biocarta, HumnaCyc, KEGG, NCI, Panther, Reactome, and SPIKE. We found that only 9236 linked genes in the package are matched with genes in our BRCA dataset.

#### Canonical correlation analysis

In our simulation study, we have demonstrated that network-based regularization utilizing network graph information can drastically improve true positive selection when correlation of linked genes is indeed higher than that of unlinked genes. Therefore, we first investigated the correlation of 9236 linked genes from BRCA dataset before conducting association analysis. From the incorporated biological network databases, we have 207,475 genetic links (edges) among 9236 genes. Since the number of CpG sites each gene ranges from 1 to 466, we computed the canonical correlation coefficient (CCC) between two linked genes which contain multivariate DNA methylation levels. Canonical correlation is a way of measuring the linear relationship between two multi-dimensional variables [28]. It essentially finds two sets of basis vectors such that the correlations between two projections of the multi-dimensional variables onto these basis vectors are mutually maximized. For each subtype, we obtained CCC of 207,475 paired genes. The sample mean of CCC is 0.8501 for the Basal subtype, 0.8841 for the Her2 subtype, 0.7747 for the LumA subtype and 0.84 for the LumB subtype.

In order to determine statistical significance of relationship between biologically linked genes and their canonical correlation, we performed a permutation test for each subtype. The total number of all possible pairs among *p*= 9236 genes can be computed as *p*(*p*−1)/2= 42,647,230. So, we randomly chose 207,475 pairs among 42,647,230 and computed the sample mean of CCC for the selected 207,475 pairs. This process was repeated *K* times. Let us denote the sample mean of CCC for the *k*-th permuted pairs by *c*_*k*_, the permutation *p*-value can then be computed as

$$p\text{-value} = \sum_{k=1}^K \frac{I(c_k > c^*) + 1}{K+1}, $$

where *c*^∗^ is the sample mean of CCC from the original gene pairs. We fixed the total number of permutation as *K*= 100,000 for all subtypes. After 100,000 permutations, we computed both min*kc*_*k*_ and max*kc*_*k*_ for each subtype. In other words, the mean of CCC of permuted pairs ranges from 0.8243 to 0.8271 for the Basal subtype, from 0.8665 to 0.8691 for the Her2 subtype, from 0.7497 to 0.7527 for the LumA subtype and from 0.8185 to 0.8215 for the LumB subtype. Since max*kc*_*k*_ is less than *c*^∗^ for all of four subtypes, their permutation *p*-values are less than 10 ^-6^. The histograms of the sample mean of CCC for the permuted pairs and the original pairs are displayed in Additional file 6.

The total number of ways to choose 207,475 pairs among 42,647,230 is exceedingly large (approximately 10 ^569,756^). Although the number of permutation of 100,000 is an extremely small number compared with this value, the mean value of CCC for any permutation sets failed to exceed the mean of CCC for the original pairs. Therefore, we are certain that the correlations of DNA methylation levels among biologically linked genes are relatively high, compared with the correlations between randomly chosen gene pairs where only 0.0486% pairs are biologically linked with each other. For this reason, the network-based regularization method that can utilize the information of 207,475 genetic pairs should be applied to the BRCA dataset.

#### Genetic association analysis

**Fig. 4:**
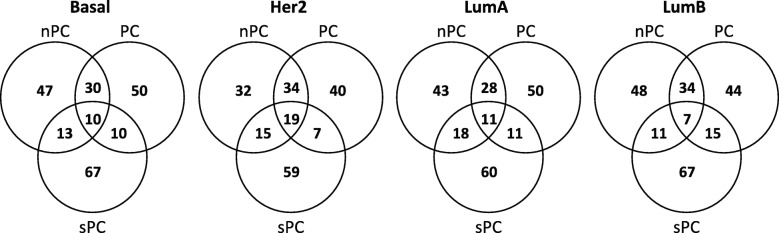
The top ranked 100 genes selected by the network-based regularization method combined with principal components (PC), normalized principal components (nPC), and supervised principal components (sPC) are summarized in the Venn diagrams for each of four breast invasive carcinoma subtypes. This analysis includes only 9236 biologically linked genes

Although our BRCA dataset has a total number of 19,296 genes, only 9236 genes are matched with the seven incorporated genetic network databases. So, we performed two different analysis. The first analysis includes only the matched 9236 genes where all genes have at least one genetic link. The second analysis includes all of 19,296 genes where 10,060 genes are isolated genes. We applied the network-based regularization method using three data dimension reduction techniques such as Net+PC, Net+nPC and Net+sPC for each BRCA subtype, since these three methods showed relatively strong true positive selection performance in our simulation studies. For each subtype of both analysis, we selected top 100 genes by selection probabilities of three methods. The number of overlapped genes in the first analysis are summarized in the Venn diagrams in Fig. 4. The result of the second analysis are summarized in the Venn diagrams in Additional file 7. We focused on these overlapped genes in the top 100 list selected by all of three methods. The number of overlapped genes are 10 for the Basal subtype, 19 for the Her2 subtype, 11 for the LumA subtype, and 7 for the LumB subtype in the first analysis, and they are 9 for the Basal subtype, 21 for the Her2 subtype, 10 for the LumA subtype, and 9 for the LumB subtype in the second analysis. These gene names and their selection probabilities are displayed in Additional file 8 for the first analysis and Additional file 9 for the second analysis.

For the Basal subtype, we identified a total of 14 genes from the first and second analysis, where 6 genes have been reported to be associated with cancers. Genes MIR124-2 [29], PBX1 [30], SKI [31], GHSR [32] and RBPMS [33] were reported to be associated with breast cancer, and a gene CYP19A1 [34] was reported to be be associated with endometrial cancer. For the Her2 subtype, 34 genes were selected by three methods from both analysis. Among them, 12 genes were reported to be associated with cancers. Four genes AQP1 [35], LFNG [36], RASSF2 [37] and WWP2 [38] were reported to be associated with breast cancer. Three genes C1orf114 [39], PRAC [40] and SPP2 [41] were reported to be associated with prostate cancer. OPRM1 [42] and GNG7 [43] were reported to be associated with oesophageal cancer and pancreatic cancer, respectively. Genes SLC2A2 [44], TNC1 [45] and MIR518A2 [46] were reported to be associated with lung cancer, gastric cancer and colorectal cancer, respectively. For the LumA subtype, a total of 18 genes were selected by three methods from both analysis, where 8 genes were reported to be associated with cancers. Genes SIAH2 [47], CDH5 [48] and HS3ST2 [49] were reported to be associated with breast cancer. Genes WNT11 [50] and THPO [51] were reported to be associated with ovarian cancer and colorectal cancer, respectively. Genes C1orf114 [39], CA3 [52] and KRT4 [53] were reported to be associated with prostate cancer, hepatocellular carcinoma and esophageal squamous cell carcinoma, respectively. For the LumB type, we identified 13 genes from both analysis. Among them, 5 genes were reported to be associated with cancers. Genes AHCYL2 [54] and PSPN [55] were reported to be associated with lung cancer. MSI2 [56], MACC1 [57] and TAGLN [58] were reported to be associated with ovarian cancer, colorectal cancer and esophageal cancer, respectively.

**Fig. 5:**
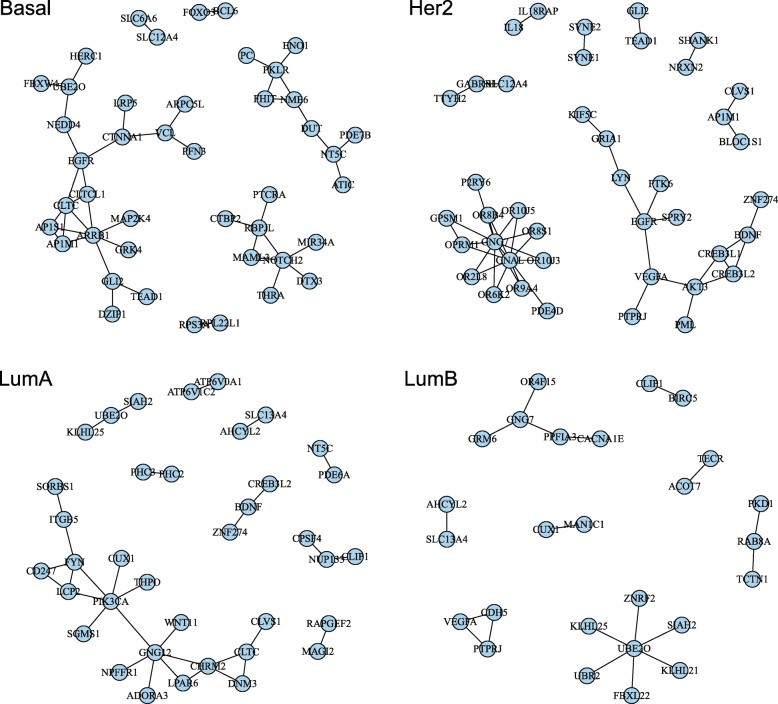
Subnetworks of the seven incorporated biological network databases among top ranked 100 genes selected by the network-based regularization method combined with normalized principal components are displayed for each of four breast invasive carcinoma subtypes. Isolated genes are not shown

Next, for each subtype we constructed the subnetwork of top ranked 100 genes selected by the network-based regularization combined with the normalized principal component based on the seven incorporated biological network databases. Figure 5 displays only linked genes among top ranked 100 genes, where 43 genes for the Basal subtype, 41 genes for the Her2 subtype, 37 genes for the LumA subtype and 26 genes for the LumB subtype have genetic links. In the Basal subtype, the subnetwork contains 6 liked genes (CTBP2, DTX3, MAML3, NOTCH2, PTCRA and RBPJL) from Notch signaling pathway on the KEGG database. Also, it contains 6 linked genes (AP1M1, AP1S1, ARRB1, CLTC, CLTCL1 and EGFR) from both Membrane trafficking and Vesicle-mediated transport pathways on the Reactome database. In the Her2 subtype, the subnetwork contains 13 linked genes (GNAL, GNG7, GPSM1, OPRM1, OR10J3, OR10J5, OR2L8, OR6K2, OR8B4, OR8S1, OR9A4, P2RY6 and PDE4D) from G protein-coupled receptors (GPCRs) signaling pathway on the Reactome database. In the LumA subtype, the subnetwork also contains 5 linked genes (ADORA3, CHRM2, GNG12, LPAR6 and NPFFR1) from G protein-coupled receptors (GPCRs) signaling pathway on the Reactome database. In the LumB subtype, the subnetwork contains 7 linked genes (FBXL22, KLHL21, KLHL25, SIAH2, UBE2O, UBR2 and ZNRF2) from Adaptive immune system, Antigen processing: Ubiquitination & Proteasome degradation and Class I MHC mediated antigen processing & presentation pathways on the Reactome database. The proposed approach was able to identify potentially cancer-related genetic pathways as well as cancer-related genes, utilizing the incorporated 7 geneticnetwork databases.

## Conclusions

In this article, we have proposed new variable selection approach to utilize prior biological network information for analysis of high-dimensional DNA methylation array data. Most of existing statistical methods for case-control association studies with DNA methylation data are not designed to use prior biological network information such as genetic pathways and signaling pathways, although DNA methylation levels between biologically linked genes are highly correlated with each other. The proposed approach is first to capture gene level signals from multiple CpG sites using a dimension reduction technique like normalized principal components and then to perform network-based regularization based on biological network graph information. In our simulation studies, we demonstrated that the proposed selection approach outperforms other statistical methods that ignore genetic network structures in terms of true positive rates. We also applied it to breast cancer data consisting of 450K DNA methylation array data, where the proposed approach was able to select potentially cancer-related genes and genetic pathways.

In our simulation and data analysis, we applied four different dimension reduction techniques. Surprisingly, we found that selection performance of four techniques were quite different from each other even if the same network-based regularization method was performed. In particular, the number of overlapped genes in top 100 lists created by different reduction techniques is relatively small in analysis of breast cancer data. This result indicates that gene-level features of four different reduction techniques are generated in quite a different way. Specifically, both supervised principal components and partial least squares aim to find features that associated with a phenotype outcome, where the former selects significant CpG sites while the later weights estimated regression coefficients. Although both principal components and normalized principal components find features that have the largest variance, normalizing makes a difference between two components. Based on true positive selection in simulation studies, we concluded that the normalized principal component is the most appropriate among four techniques for dimension reduction of high-dimensional DNA methylation data. However, we believe that selection performance of network-based regularization can be improved if we can generate new gene-level features that include more CpG site-level information.

One practical issue in the application of the proposed approach to high-dimensional DNA methylation data is to determine which existing biological networks to use and how to account for their uncertainty. Although we incorporated seven biological network databases to apply our breast cancer data, we could focus on the specified biological networks such as the known cancer-related genetic pathways and the large-scale protein-protein interaction network. However, many genes can be unnecessarily excluded in the analysis if we limit to genes within particular genetic pathways. In our example, we had only 9236 genes matched with our incorporated biological network databases among 19,296 genes. Since research on genetic network is steadily growing and biological network databases are periodically updated, the proposed approach will be more useful to precisely identify cancer-related genes and genetic pathways in the near future.

The proposed approach can perform both pathway-level and gene-level selection. However, DNA methylation data consists of three layers which are pathways, genes and CpG sites. There currently exist no methods that simultaneously perform three level selection, i.e., cancer-related pathways, outcome-related genes within the selected pathways, causal CpG sites within the selected genes. Most of existing statistical methods for case-control association studies are designed to select only causal CpG sites, only outcome-related genes or both. We think that development of new statistical model that can capture all of three level signals is next stage for analysis of DNA methylation data. Although the proposed approach has a limitation to select causal CpG within outcome-related genes, we suggested new paradigm to perform both pathway-level and gene-level selection in DNA methylation analysis. So, we believe that the proposed approach can be extended to the model that performs three level selection in the future.

## Materials and methods

Let us denote the methylation values of the *m*-th gene by $\phantom {\dot {i}\!}X_{m}=({\boldsymbol x}_{1}, {\boldsymbol x}_{2}, \ldots, {\boldsymbol x}_{k_{m}})^{\mathrm {T}}$, where ***x***_*j*_=(*x*_1*j*_,*x*_2*j*_,…,*x*_*nj*_)^T^ is the *n*-dimensional vector representing the methylation levels of the *j*-th CpG site for *n* individuals, and *k*_*m*_ is the total number of CpG sites in the *m*-th gene. Note that some small genes can have only 1 CpG site while big genes have hundreds of CpG sites. The total number of CpG sites is $\sum _{m=1}^{p} k_{m}$ when we consider *p* genes in the analysis. Without loss of generality, we assume that *X*_*m*_ is a mean-centered matrix, i.e, $\sum _{i=1}^{n} x_{ij}=0$ for all *j*=1,…,*k*_*m*_. Here, we focus on a case-control association study, so the outcome *y*_*i*_=1 if the *i*-th individual is a case while *y*_*i*_=0 if the *i*-th individual is a control.

### Dimension reduction techniques

Principal component analysis (PCA) is one of the most popular dimension reduction techniques. It aims to find weighted linear combinations of original predictors. The first PC of the *m*-th gene can be written as

$${\boldsymbol z}_{m}^{\text{PC}} = X_{m}{\boldsymbol \theta}, $$

where the weight vector $\phantom {\dot {i}\!}{\boldsymbol \theta }=(\theta _{1},\ldots,\theta _{k_{m}})^{\mathrm {T}}$ is estimated so that ${\boldsymbol z}_{m}^{\text {PC}}$ can have the largest variance subject to the constraint that $\|{{\boldsymbol \theta }}\|_{2}^{2}=1$, where ∥·_2_∥ is a *l*_2_ norm. This is equivalent to the first eigenvector of the covariance matrix of *X*_*m*_. We also define the first normalized PC (nPC) of the *m*-th gene as

$${\boldsymbol z}_{m}^{\text{nPC}}=\frac{1}{\sqrt{e}}z_{m}^{\text{PC}}, $$

where *e* is the first eigenvalue of the covariance matrix of *X*_*m*_. The nPC is frequently used in analysis of signal processing, which is also known as a whitening process [59]. Projecting DNA methylation levels onto the principal components can remove the second-order linear correlations and perform dimension reduction by discarding dimensions with low variances. In addition to decorrelation, the nPC normalizes the variance in each dimension so that all dimensions have unit variance. Geometrically, this makes the data to be rotationally symmetric just like a sphere. Therefore, $\|{{\boldsymbol z}_{m}^{\text {nPC}}}\|_{2}=1$.

While both PC and nPC can be extracted without using a phenotype outcome, supervised PC (sPC) [60, 61] and partial least square (PLS) [62] capture a gene level signal based on phenotypic associations with DNA methylation levels. The sPC first investigates an association strength between individual CpG sites and a phenotype outcome. It then selects CpG sites whose association signals are greater than an optimally chosen threshold. Finally, PCA is applied to the selected CpG sites. Similar to PC, the first component of sPC can be written as

$${\boldsymbol z}_{m}^{\text{sPC}} = \tilde{X}_{m}{\boldsymbol \theta}, $$

where $\tilde {X}_{m}=({\boldsymbol x}_{1}, {\boldsymbol x}_{2}, \ldots, {\boldsymbol x}_{q_{m}})^{\mathrm {T}}$ and $\phantom {\dot {i}\!}{\boldsymbol \theta }=(\theta _{1},\ldots,\theta _{q_{m}})^{\mathrm {T}}$ if *q*_*m*_ CpG sites in the *m*-th gene are selected. The PLS basically finds the best orthogonal linear combinations of DNA methylation levels for predicting a phenotype outcome. Similar to sPC, it first estimates a regression coefficient of simple logistic regression between a CpG site and a phenotype outcome. Let us denote the regression coefficient of the *j*-th CpG site by $\hat {\gamma }_{j}$ and then the coefficient vector $\hat {\boldsymbol \gamma }=(\hat {\gamma }_{1},\hat {\gamma }_{2},\ldots,\hat {\gamma }_{k_{m}})^{\mathrm {T}}$. Next, the weight vector is computed as normalizing the coefficient vector which is divided by the squared *l*_2_-norm of the coefficient vector, i.e., ${\boldsymbol \theta }=\hat {\boldsymbol \gamma }/\|{\hat {\boldsymbol \gamma }}\|_{2}$. Then, the first component of PLS can be defined as

$${\boldsymbol z}_{m}^{\text{PLS}} = \frac{X_{m}{\boldsymbol \theta}}{{\boldsymbol \theta}^{\mathrm{T}} {\boldsymbol \theta}}. $$

Using the first component from one of these four dimension reduction techniques, methylation levels at the *k*_*m*_-dimensional CpG sites of the *m*-th gene can be replaced by one-dimensional feature. Consequently, $\sum _{m=1}^{p} k_{m}$ CpG sites are reduced down to *p* gene-level features as we apply dimension reduction to each of genes. These features can be matched with the *p*-dimensional Laplacian matrix representing a network structure. Let us denote the feature of the *i*-individual and the *m*-th gene by *z*_*im*_ and *z*_*i*_=(*z*_*i*1_,…,*z*_*ip*_)^T^. As a result, each feature can play the role of predictors in the network-based regularization. In simulation study, the network-based regularization methods based on the features generated from four different dimension reduction techniques are compared with each other.

### Network-based regularization

The penalized logistic likelihood using network-based regularization can be written as

1 $$ \begin{aligned} {}-\frac{1}{n}\sum_{i=1}^{n} [y_{i} \log p(z_{i}) &+(1-y_{i})\log(1-p(z_{i})]\\ & + \lambda\alpha\|{{\boldsymbol \beta}}\|_{1}+ \lambda(1-\alpha)\boldsymbol{\beta}^{\mathrm{T}}S^{\mathrm{T}}LS\boldsymbol{\beta}, \end{aligned}  $$

where ∥·∥_1_ is a *l*_1_ norm, ***β***=(*β*_1_,…,*β*_*p*_)^T^ is the *p*-dimensional coefficient vector and

$$p(z_i)=\frac{\exp\left(\beta_0+z_{i}^{\mathrm{T}}{\boldsymbol \beta}\right)}{1+\exp\left(\beta_0+z_{i}^{\mathrm{T}}{\boldsymbol \beta}\right)} $$

is the probability that the *i*-th individual is a case. The tuning parameter *λ* controls sparsity of the network-based regularization, *α*∈[0,1] is a mixing proportion between lasso and graph-constrained penalties. The diagonal matrix *S*=diag(*s*_1_,…,*s*_*p*_),*s*_*u*_∈{−1,1} has the estimated signs of regression coefficients on its diagonal entries, which can be obtained from ordinary regression for *p*<*n*, and ridge regression for *p*≥*n*. It has been demonstrated that the matrix *S* can accommodate the problem of failure of local smoothness between linked genes, where two adjacent risk genes have opposite effects on a phenotype outcome when the corresponding regression coefficients have different signs [6].

In the penalized likelihood (1), the *p*-dimensional Laplacian matrix *L*={*l*_*uv*_} represents a graph structure when the network information among genes is provided. It is defined as

$$l_{uv}\,=\,\left\{ \begin{array}{ll} 1 & \text{if} \quad u=v \text{ and} d_{u}\neq0 \\ \!-(d_u d_v)^{-\frac{1}{2}} & \text{if} \quad u \text{ and } v \text{ are linked with each other} \\ 0 & \text{otherwise}, \end{array} \right. $$

where *d*_*u*_ is the total number of genetic links of the *u*-th gene. This Laplacian penalty is a combination of the *l*_1_ penalty and squared *l*_2_ penalty on degree-scaled differences of coefficients between linked genes. It induces both sparsity and smoothness with respect to the correlated or linked structure of the regression coefficients. It has been shown that a desirable grouping effect can be reached by specifying genetic links among genes in the model [1, 6].

Once we fill out the Laplacian matrix based on genetic network information, we can estimate an intercept parameter *β*_0_ and the coefficient vector ***β***, as minimizing the penalized likelihood (1) for fixed values of *α* and *λ*. This is considered as a convex optimization problem. There are relatively many statistical softwares for convex optimization of lasso-type penalty functions [8, 13, 27, 63–67]. Most of them provide the pathwise solutions to *β*_0_ and ***β*** for fixed values of *α* and *λ*. However, a practical problem is how to pick up the optimal tuning parameters *α* and *λ*. Although a cross-validation method is most commonly applied to find the optimal tuning parameters, its selection result is not stable because cross-validation is based on random split samples. Inconsistent choice of the tuning parameters leads to have either too small number of true positives or too many false positives since they essentially control the number of selected genes.

### Selection probability

As a solution to the tuning parameter problem in regularization, Meinshausen and Bühlmann [68] originally proposed to compute selection probability of individual variables from repeated half-sample resampling. They demonstrated that selection probability can produce very stable selection result, compared with variable selection using cross-validation. For this reason, it has been widely used for genetic association studies with high-dimensional data [7, 8, 27, 69, 70].

Let *I*_*s*_ be the *s*-th random subsample that has a size of ⌊*n*/2⌋ without replacement, where ⌊*x*⌋ is the largest integer not greater than *x*. If a balanced design between cases and controls is desirable, we can randomly choose ⌊*n*_1_/2⌋ cases and ⌊*n*_2_/2⌋ controls among *n* samples, where *n*_1_ and *n*_2_ are the number of cases and the number of controls, respectively. For each *α*, the pathwise solutions to regression coefficients (*β*_0_,***β***) based on the subsamples of $\phantom {\dot {i}\!}(z_{i},y_{i})_{i\in I_{s}}$ can be obtained using one of the softwares for convex optimization. We applied an R package ‘pclogit’ [8]. Let us denote the *j*-th estimated regression coefficient for fixed values of *α* and *λ* by $\hat {\beta }_{j}(I_{s};\alpha,\lambda)$. Next, we need to count the total number of $\hat {\beta }_{j}(I_{s};\alpha,\lambda)\neq 0$ for *s*=1,…,*S* where *S* is the total number of resampling. Finally, the selection probability of the *j*-th gene is computed by

$$ \text{SP}_j = \max_{\alpha,\lambda} \frac{1}{S} \sum_{s=1}^S I\left(\hat{\beta}_{j}(I_s;\alpha,\lambda)\neq0\right), $$

where *I*(·) is an indicator function. We fixed *S*=100 for simulation study and *S*=500 for real data analysis.

**Fig. 6:**
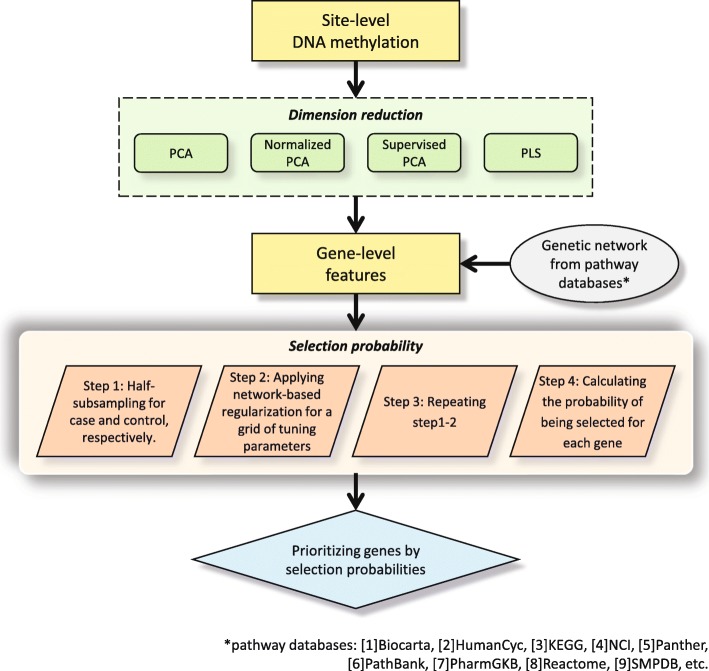
A flowchart of the proposed network-based regularization method combined with four different dimension reduction techniques

One of the great advantages of selection probability is that we do not need to select the optimal tuning parameters *α* and *λ*. We first set a fine grid value of *α* between 0 and 1 and then the pathwise solutions to $\hat {\beta _{0}}$ and $\hat {{\boldsymbol \beta }}$ along with different *λ* values can be computed for each *α*. Next, we compare selection probability for each (*α*,*λ*) and then just pick up the largest selection probability over all (*α*,*λ*). After we compute the selection probability of all *p* genes, we can prioritize genes from the largest selection probability to the smallest selection probability. A flowchart in Fig. 6 summarizes the entire procedure of the proposed network-based regularization combined with dimension reduction techniques.

Finally, we recommend to select a particular number of top-ranked genes rather than using the threshold of selection probability since selection probability is a relative measurement. Its magnitude depends on the numerical values of tuning parameters *α* and *λ*. Actually, selection result depends on *λ* rather than *α* since *λ* controls sparsity, i.e., the number of nonzero coefficients. *α* can affect the numerical values of nonzero coefficients, but computation of selection probability is based only on either selected or not selected. Indeed, overall selection probabilities of individual genes tend to be decreasing as *λ* values are increasing, regardless of the numerical value of *α*. However, ranking of genes based on their selection probabilities is rarely changed for different values of *α* and *λ*. Therefore, we can use only a few *α* values to reduce computational time, while the number of *λ* for each *α* is fixed.

## Additional files

Additional file 1 An example of four network modules used in the second scenario of the simulation study. Each network module includes 12 outcome-related genes colored in red.(PDF 91 kb)

Additional file 2 The averaged true positive rates of the network-based regularization methods combined with four different dimension reduction techniques such as principal components (Net+PC), normalized PC (Net+nPC), supervised PC (Net+sPC), partial least square (Net+PLS) and group lasso are displayed along with different number of selected genes ranked by selection probability, when the number of causal CpG sites in an outcome-related gene *ω* and the noise level *σ* have different values.(PDF 47 kb)

Additional file 3 The averaged true positive rates of the network-based regularization method combined with normalized principal component (Net+nPC), two sample *t*-test using PCA (T-test), global test (GT), SAM-GS and Hotelling’s *T*^2^ test (HT) are displayed along with different number of selected genes ranked by selection probability for Net+nPC and *p*-values for four individual tests, when the number of causal CPG sites in an outcome-related gene *ω* and the noise level *σ* have different values.(PDF 48 kb)

Additional file 4 The histograms of the number of CpG sites each gene for both simulation data generated from a Gamma distribution and breast cancer data.(PDF 5 kb)

Additional file 5 The averaged true positive rates of 4 individual tests and 4 different regularization methods are compared with those of the network-based regularization method combined with normalized principal components when the proportion of outcome-related CpG sites in a causal gene *ω*=40*%* and the noise level *σ*=2.5.(PDF 9 kb)

Additional file 6 Four histograms of the sample mean of canonical correlation coefficients for the permuted 207,475 gene pairs are shown for each subtype of breast invasive carcinoma dataset. The dotted red line indicates the sample mean of canonical correlation coefficients for the original 207,475 gene pairs from incorporated 7 genetic network databases.(PDF 41 kb)

Additional file 7 The top ranked 100 genes selected by the network-based regularization method combined with principal components (PC), normalized principal components (nPC), and supervised principal components (sPC) are summarized in the Venn diagrams for each of four breast invasive carcinoma subtypes. This analysis includes 9236 biologically linked genes and 10,060 isolated genes.(PDF 27 kb)

Additional file 8 For each subtype of breast invasive carcinoma, overlapped genes among top 100 genes selected by three methods (Net+PC, Net+nPC, Net+sPC) are listed along with their selection probability (SP) computed by Net+nPC. This analysis includes only 9236 biologically linked genes.(PDF 140 kb)

Additional file 9 For each subtype of breast invasive carcinoma, overlapped genes among top 100 genes selected by three methods (Net+PC, Net+nPC, Net+sPC) are listed along with their selection probability (SP) computed by Net+nPC. This analysis includes 9236 biologically linked genes and 10,060 isolated genes.(PDF 160 kb)

## Data Availability

The datasets and R codes used in the manuscript are available from the corresponding author on reasonable request.
