# Supplementary figures and images for: Incorporating genetic networks into case-control association studies with high-dimensional DNA methylation data
Source: BMC Bioinformatics. 2019 Oct 22;20:510. doi: 10.1186/s12859-019-3040-x (PMC6805595; doi:10.1186/s12859-019-3040-x)

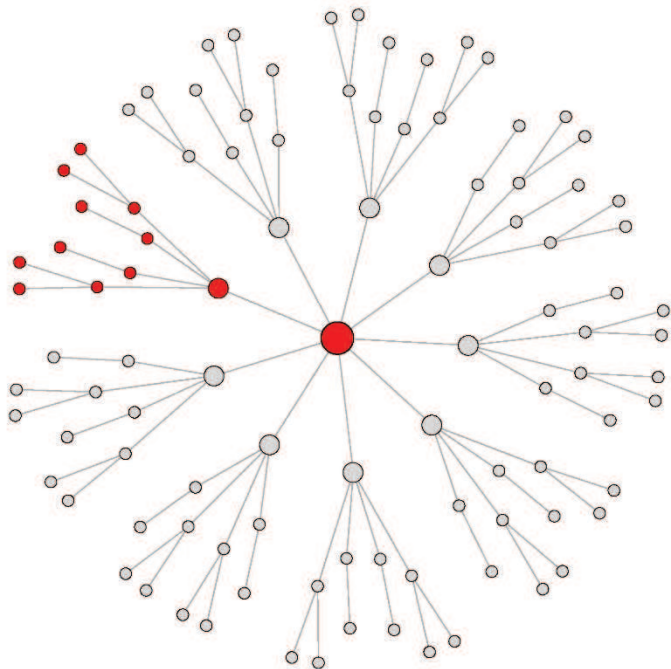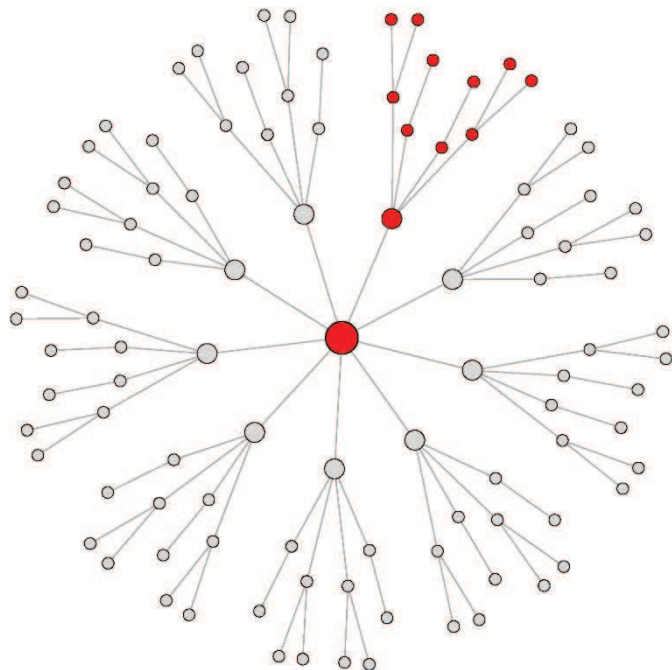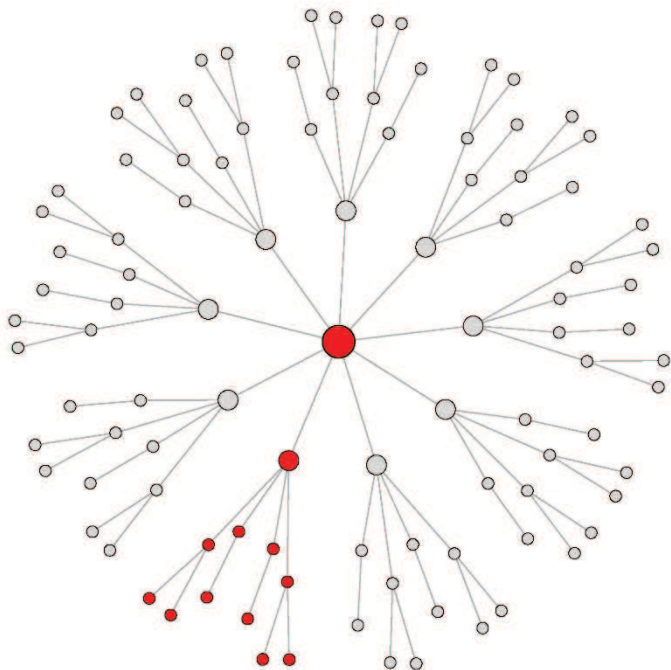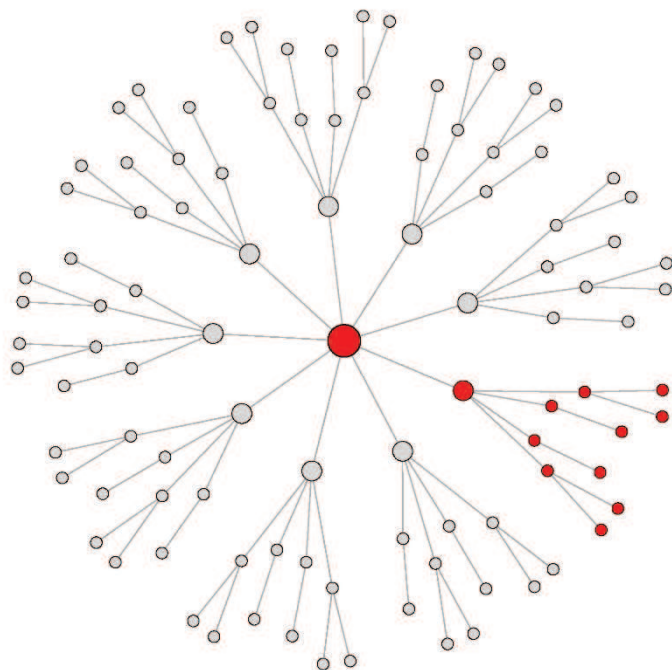

Supplement: Supplementary file 1 — An example of four network modules used in the second scenario of the simulation study. Each network module includes 12 outcome-related genes colored in red.(PDF 91 kb) [file 12859_2019_3040_MOESM1_ESM.pdf]

True positive rates

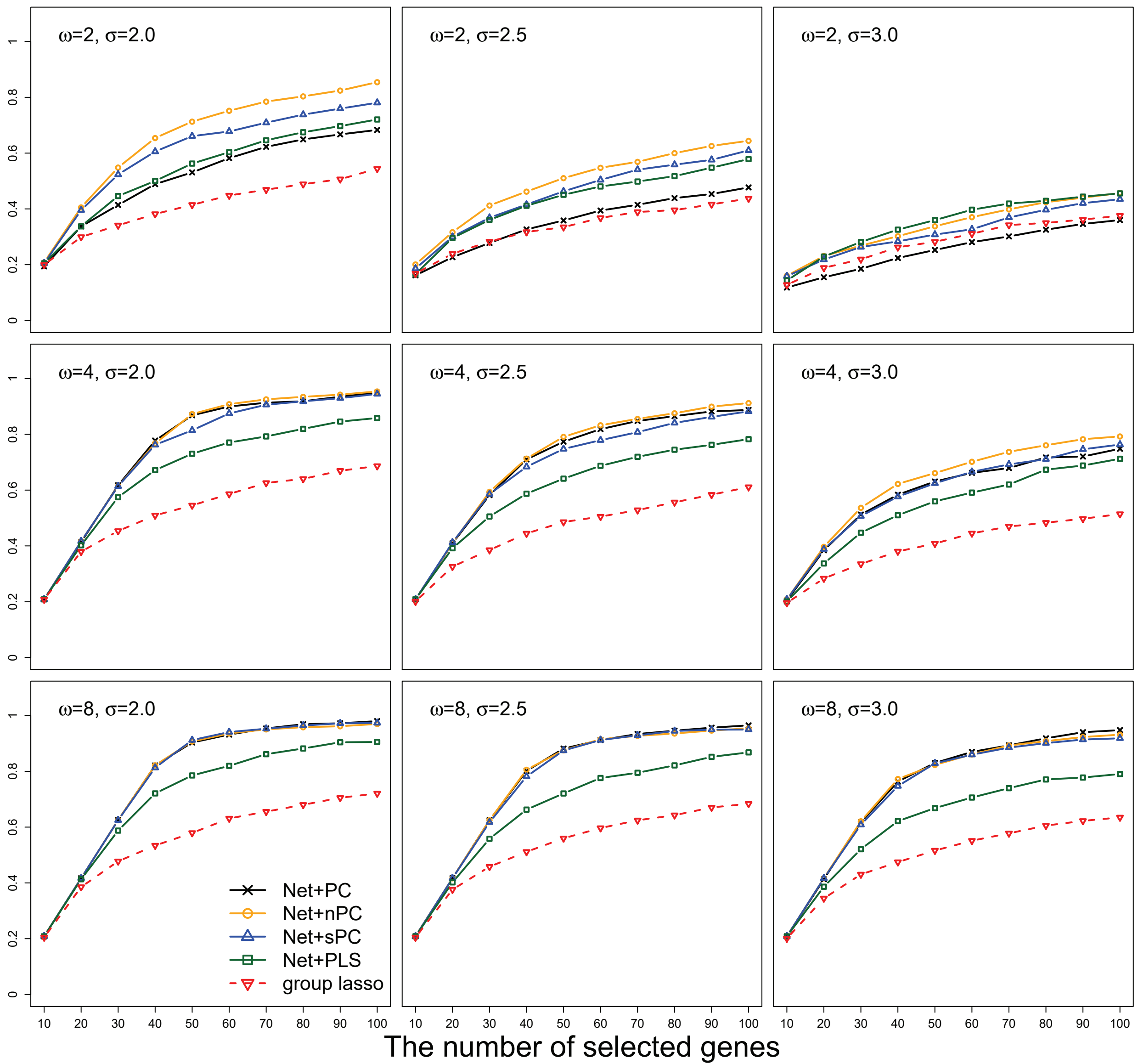

Supplement: Supplementary file 2 — The averaged true positive rates of the network-based regularization methods combined with four different dimension reduction techniques such as principal components (Net+PC), normalized PC (Net+nPC), supervised PC (Net+sPC), partial least square (Net+PLS) and group lasso are displayed along with different number of selected genes ranked by selection probability, when the number of causal CpG sites in an outcome-related gene ω and the noise level σ have different values.(PDF 47 kb) [file 12859_2019_3040_MOESM2_ESM.pdf]

True positive rates

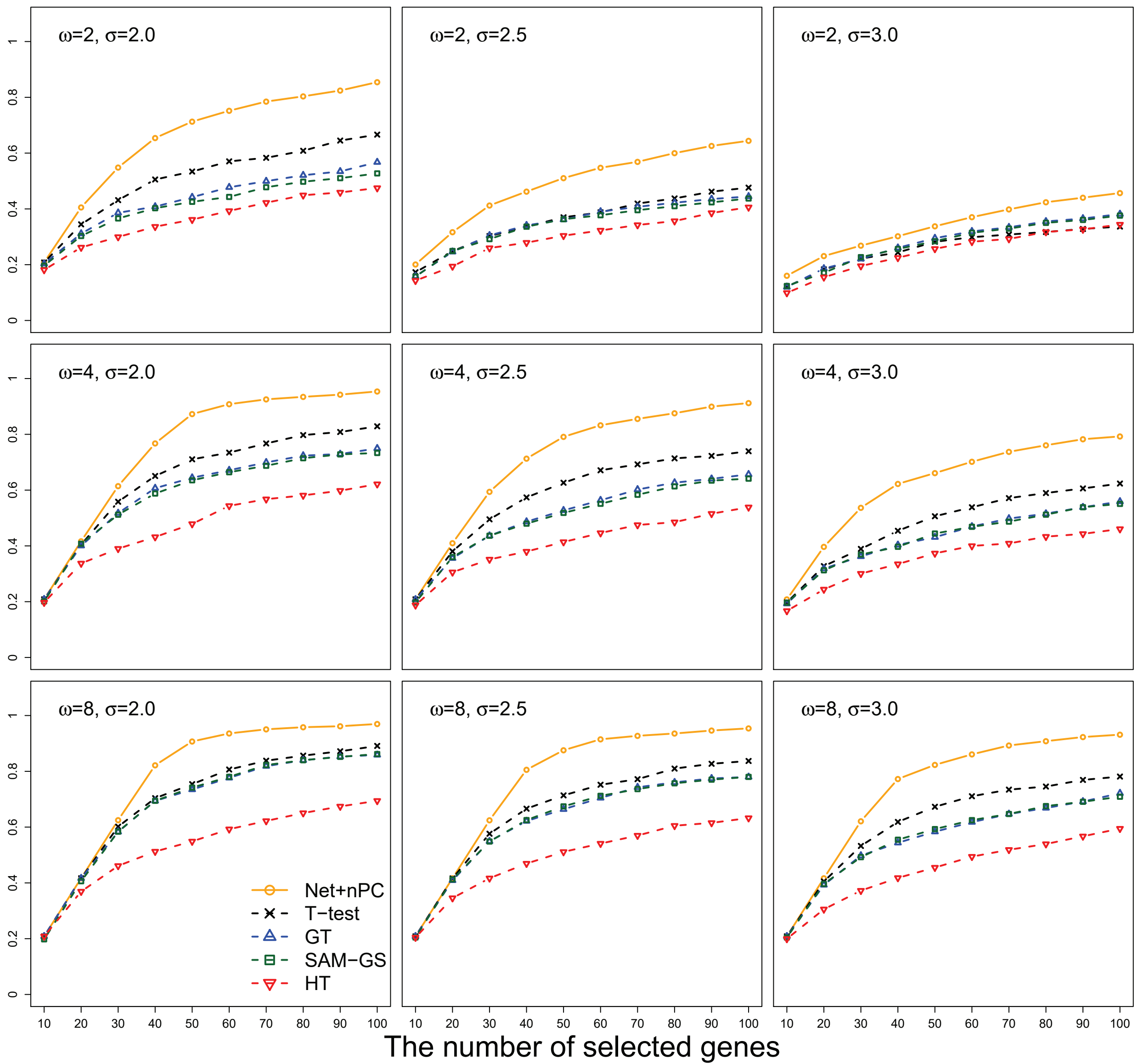

Supplement: Supplementary file 3 — The averaged true positive rates of the network-based regularization method combined with normalized principal component (Net+nPC), two sample t-test using PCA (T-test), global test (GT), SAM-GS and Hotelling’s T2 test (HT) are displayed along with different number of selected genes ranked by selection probability for Net+nPC and p-values for four individual tests, when the number of causal CPG sites in an outcome-related gene ω and the noise level σ have different values.(PDF 48 kb) [file 12859_2019_3040_MOESM3_ESM.pdf]

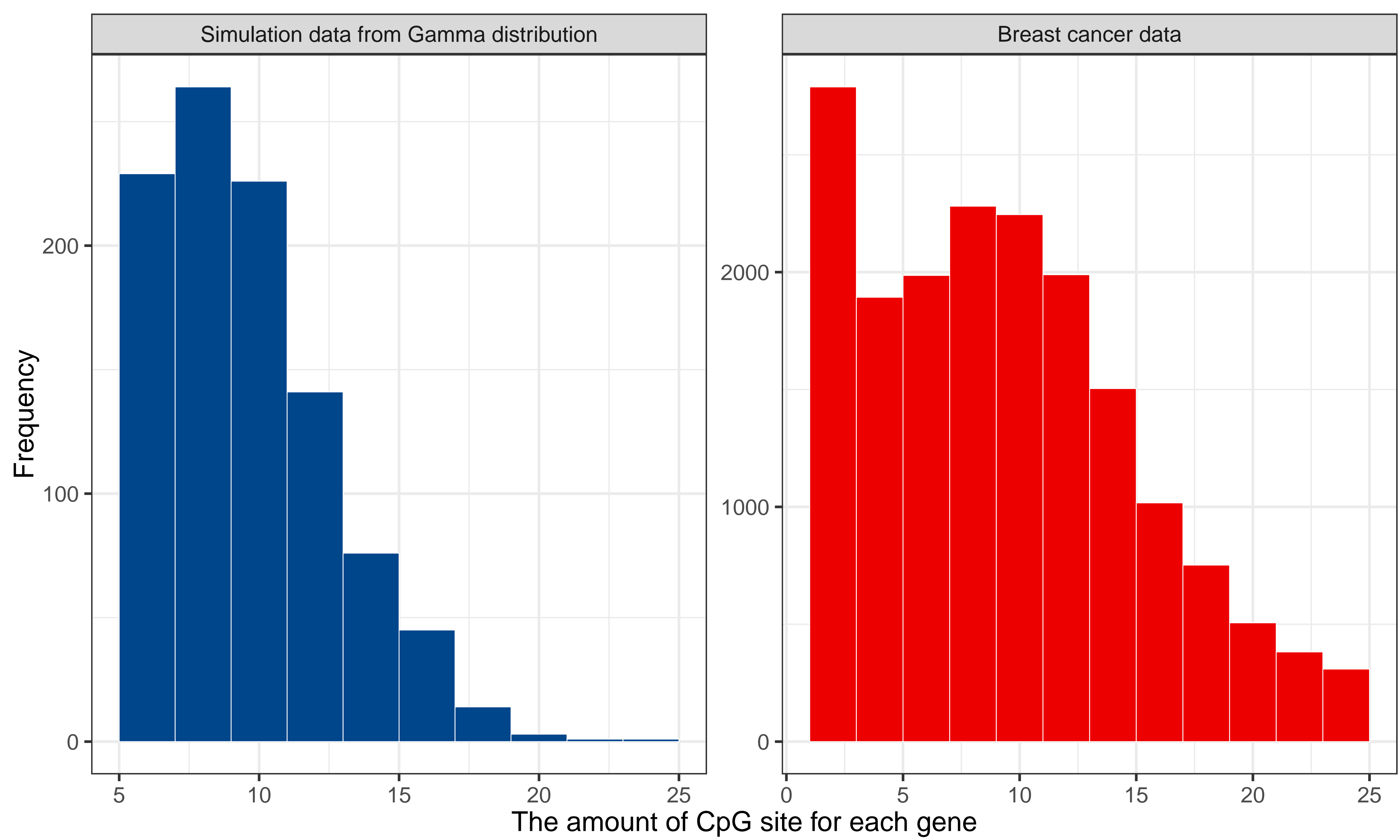

Supplement: Supplementary file 4 — The histograms of the number of CpG sites each gene for both simulation data generated from a Gamma distribution and breast cancer data.(PDF 5 kb) [file 12859_2019_3040_MOESM4_ESM.pdf]

True positive rates

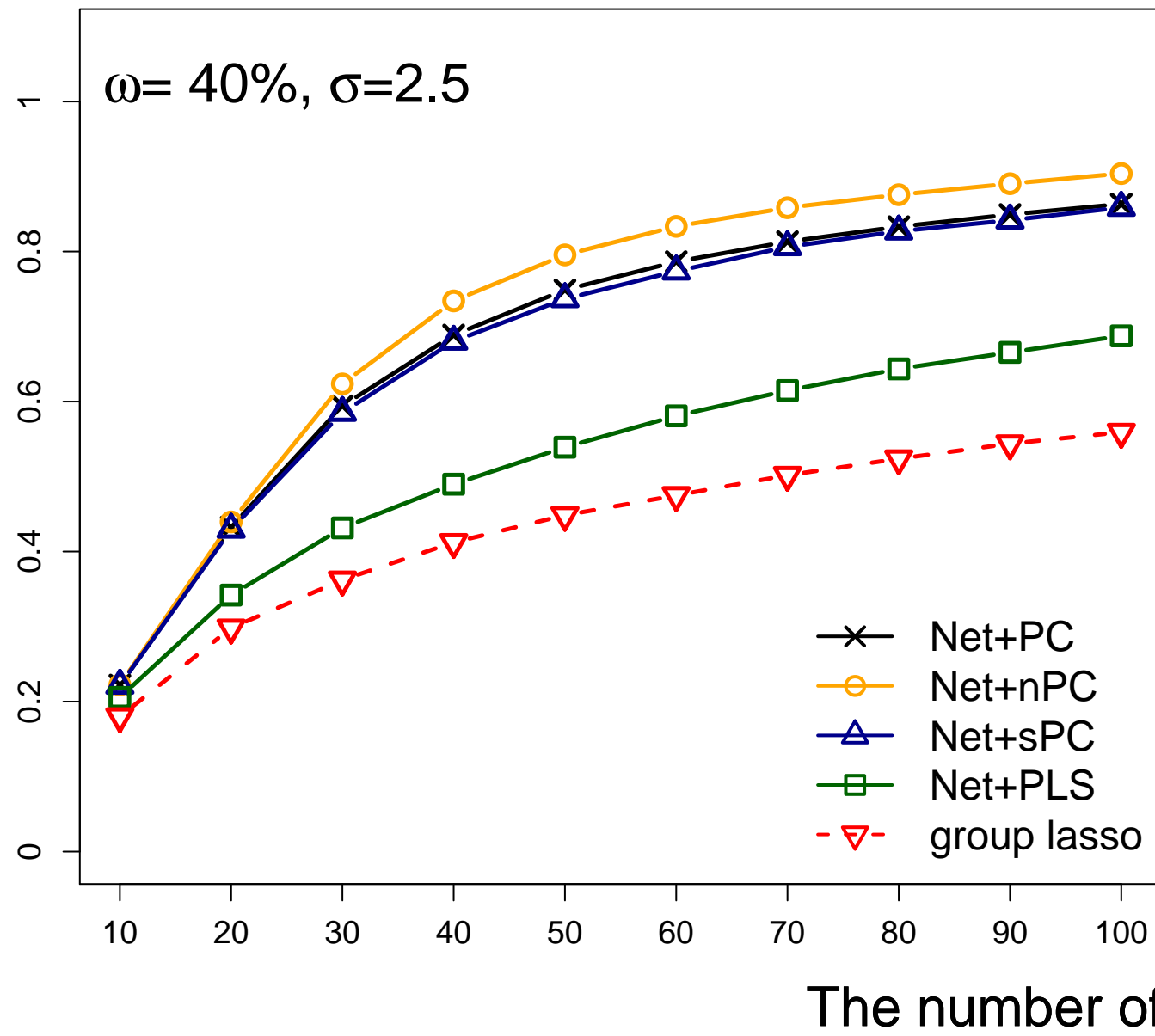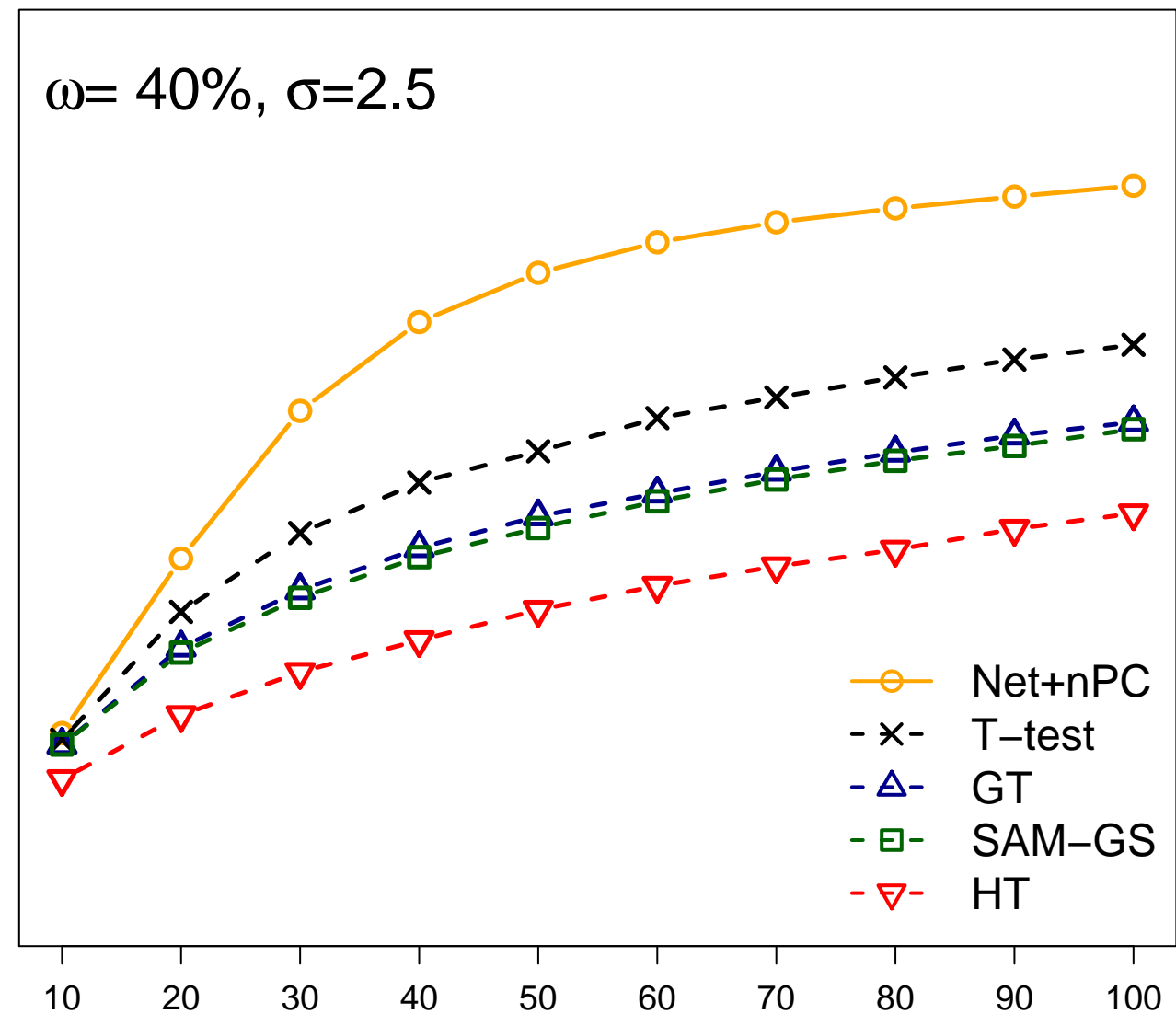

Supplement: Supplementary file 5 — The averaged true positive rates of 4 individual tests and 4 different regularization methods are compared with those of the network-based regularization method combined with normalized principal components when the proportion of outcome-related CpG sites in a causal gene ω=40% and the noise level σ=2.5.(PDF 9 kb) [file 12859_2019_3040_MOESM5_ESM.pdf]

**Basal**

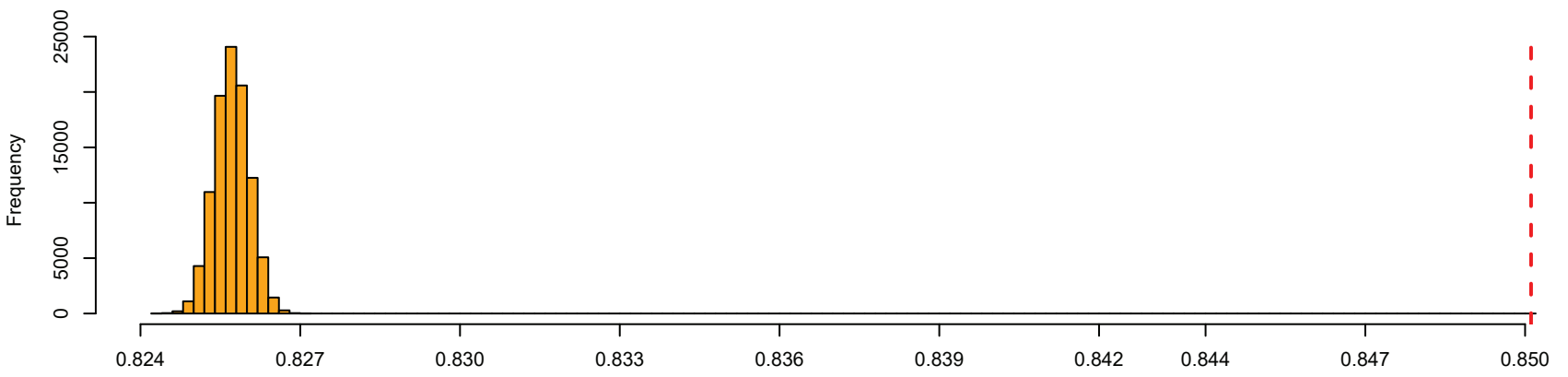

**Her2**

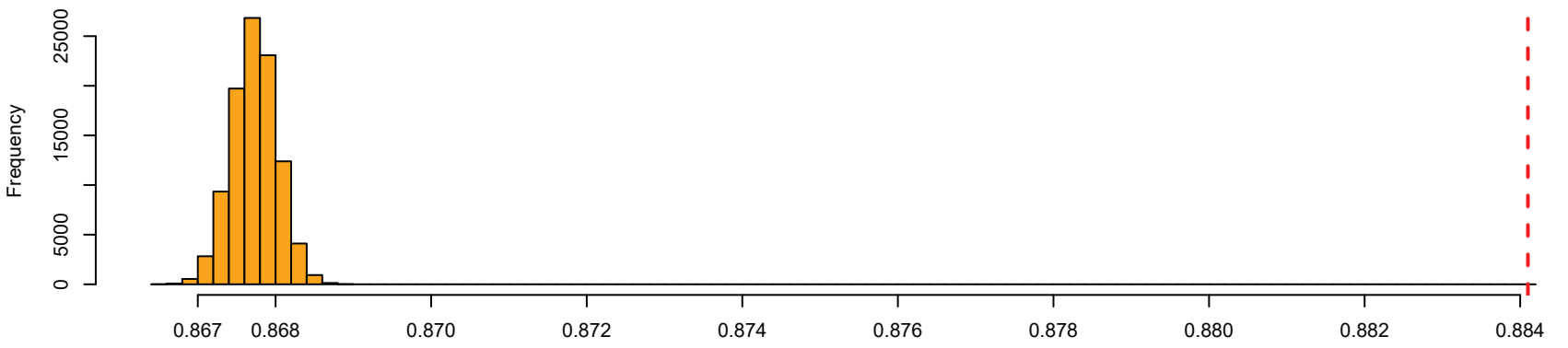

**LumA**

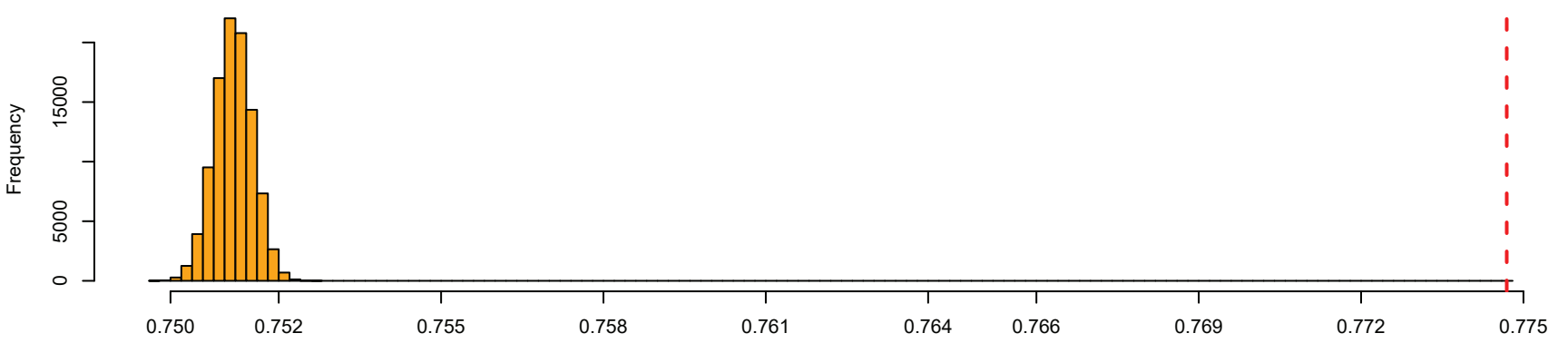

**LumB**

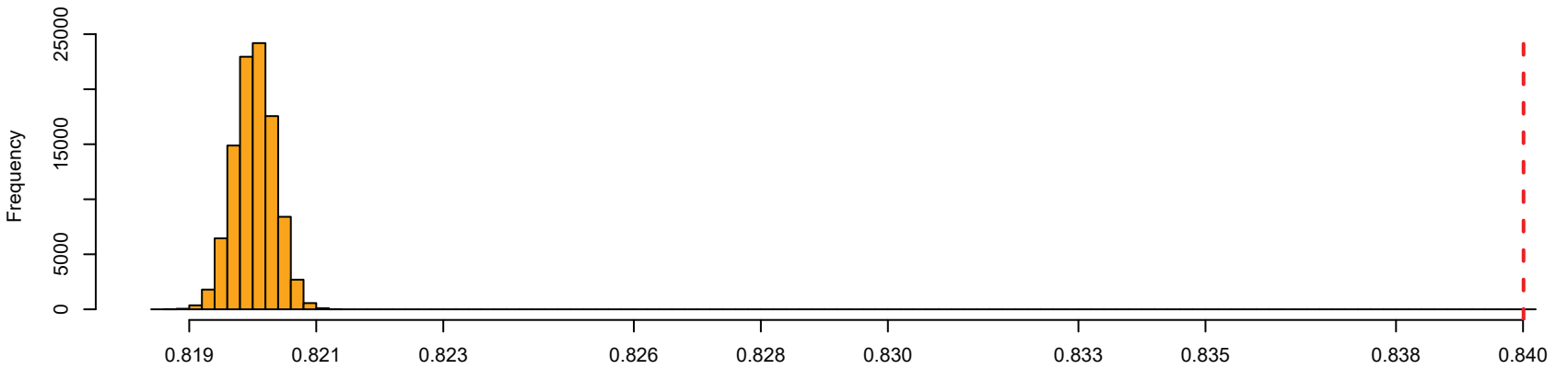

Supplement: Supplementary file 6 — Four histograms of the sample mean of canonical correlation coefficients for the permuted 207,475 gene pairs are shown for each subtype of breast invasive carcinoma dataset. The dotted red line indicates the sample mean of canonical correlation coefficients for the original 207,475 gene pairs from incorporated 7 genetic network databases.(PDF 41 kb) [file 12859_2019_3040_MOESM6_ESM.pdf]

## Basal

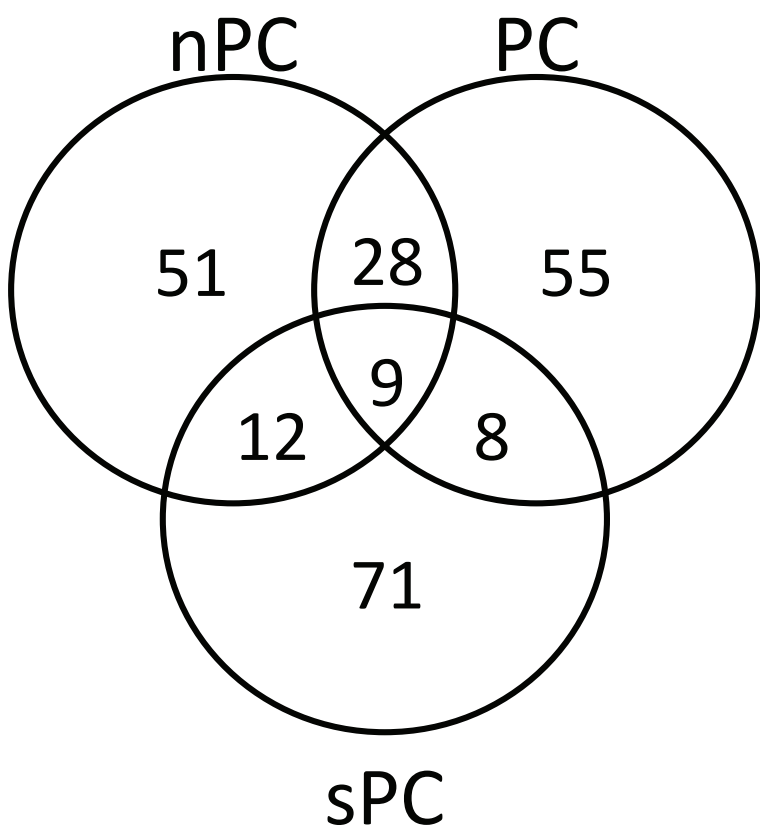

## Her2

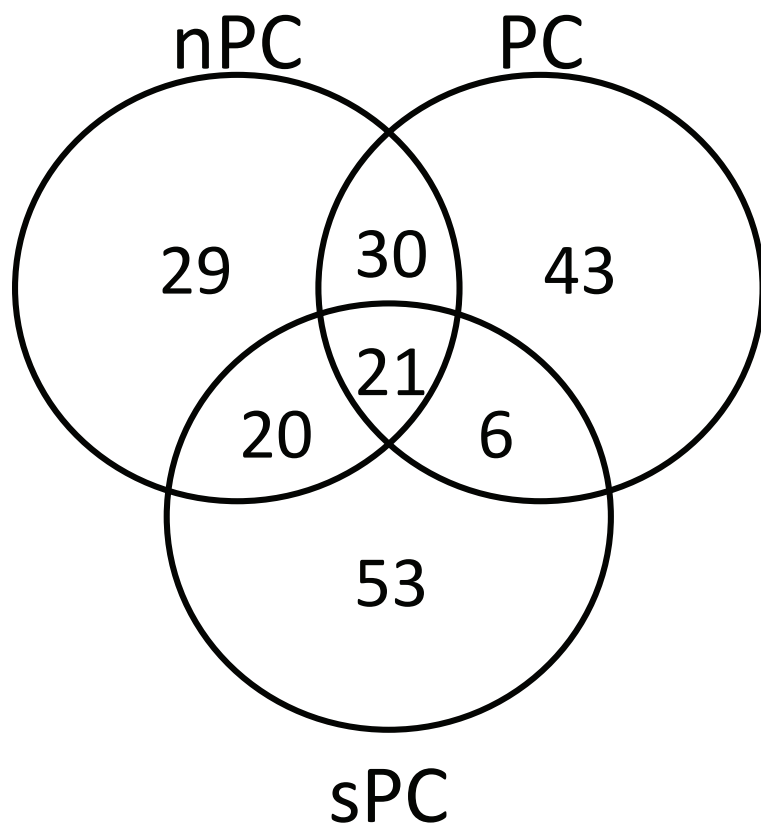

## LumA

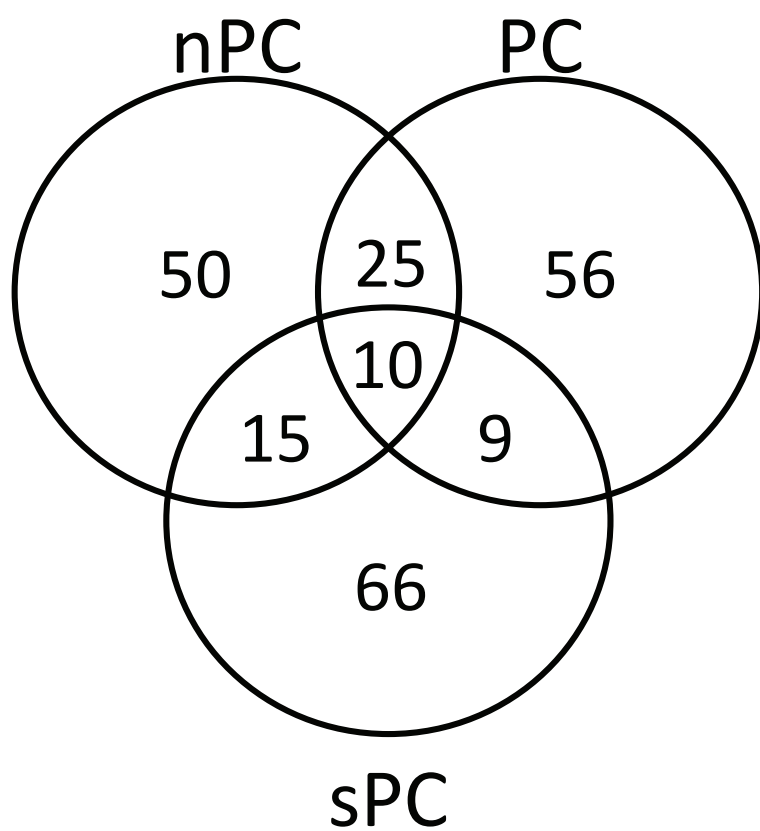

## LumB

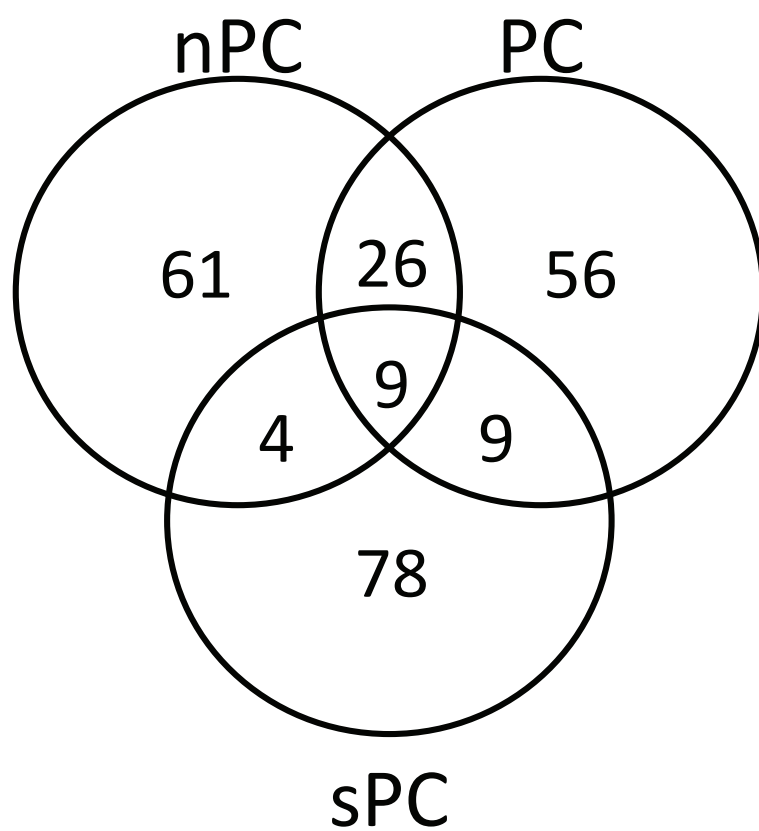

Supplement: Supplementary file 7 — The top ranked 100 genes selected by the network-based regularization method combined with principal components (PC), normalized principal components (nPC), and supervised principal components (sPC) are summarized in the Venn diagrams for each of four breast invasive carcinoma subtypes. This analysis includes 9236 biologically linked genes and 10,060 isolated genes.(PDF 27 kb) [file 12859_2019_3040_MOESM7_ESM.pdf]
