# Supplementary material for: Incorporating genetic networks into case-control association studies with high-dimensional DNA methylation data
Source: BMC Bioinformatics. 2019 Oct 22;20:510. doi: 10.1186/s12859-019-3040-x (PMC6805595; doi:10.1186/s12859-019-3040-x)
Supplement: Supplementary file 8 — For each subtype of breast invasive carcinoma, overlapped genes among top 100 genes selected by three methods (Net+PC, Net+nPC, Net+sPC) are listed along with their selection probability (SP) computed by Net+nPC. This analysis includes only 9236 biologically linked genes.(PDF 140 kb) [file 12859_2019_3040_MOESM8_ESM.pdf]

Basal		Her2		LumA		LumB	
gene	SP	gene	SP	gene	SP	gene	SP
CYP19A1	1	GNG7	1	CA3	1	AHCYL2	1
PFN3	1	OPRM1	1	CLIP1	1	APCS	1
SLC25A2	1	OR10J5	1	MYLK	1	PSPN	1
GHSR	0.994	OR8B4	1	RYR3	1	TAGLN	1
GPR37L1	0.994	SLC2A2	1	SIAH2	1	TAS1R1	1
MIR124-2	0.994	NLRP3	0.996	CDH5	0.998	OR4F15	0.994
PTCRA	0.99	OR10J3	0.996	HS3ST2	0.996	MSI2	0.972
KIF24	0.986	OR2L8	0.996	CHST3	0.986		
PBX1	0.918	RORA	0.99	THPO	0.982		
SKI	0.896	A1CF	0.986	ATP6V1C2	0.98		
		AQP1	0.984	WNT11	0.98		
		TCN1	0.972				
		BEGAIN	0.97				
		LFNG	0.962				
		SHANK1	0.942				
		RASSF2	0.926				
		IL21	0.912				
		ZNF274	0.888				
		CDH10	0.87				
