# Supplementary material for: Incorporating genetic networks into case-control association studies with high-dimensional DNA methylation data
Source: BMC Bioinformatics. 2019 Oct 22;20:510. doi: 10.1186/s12859-019-3040-x (PMC6805595; doi:10.1186/s12859-019-3040-x)
Supplement: Supplementary file 9 — For each subtype of breast invasive carcinoma, overlapped genes among top 100 genes selected by three methods (Net+PC, Net+nPC, Net+sPC) are listed along with their selection probability (SP) computed by Net+nPC. This analysis includes 9236 biologically linked genes and 10,060 isolated genes.(PDF 160 kb) [file 12859_2019_3040_MOESM9_ESM.pdf]

Basal		Her2		LumA		LumB	
gene	SP	gene	SP	gene	SP	gene	SP
LOC134466	1	C1orf114	1	C1orf133	1	ANKRD45	1
SLC25A2	1	GNG7	1	CA3	1	LRRN3	1
PFN3	0.998	LOC254312	1	FAM18A	1	RILPL1	1
RBPMS	0.992	MIR921	1	C1orf114	0.998	TAGLN	1
GHSR	0.984	OPRM1	1	KRT4	0.994	TAS1R1	1
GPR37L1	0.968	OR8B4	1	SLC7A14	0.994	ZFP106	1
POPDC2	0.944	WWP2	1	ZNF80	0.994	C1orf133	0.99
MIR124-2	0.942	SPP2	0.996	CDH5	0.984	APCS	0.966
LRRN3	0.878	LRRC31	0.994	EGFLAM	0.984	MACC1	0.958
		SLC2A2	0.994	SIAH2	0.974		
		LOC399959	0.992				
		OR2L8	0.992				
		DPP6	0.988				
		MTERFD2	0.974				
		OR10J5	0.97				
		KRTAP20-4	0.946				
		ZNF80	0.944				
		MIR518A2	0.94				
		C1QTNF4	0.934				
		ANKRD45	0.906				
		PRAC	0.892				
